# Supplementary material for: Estimating the strength of selection for new SARS-CoV-2 variants
Source: Nat Commun. 2021 Dec 14;12:7239. doi: 10.1038/s41467-021-27369-3 (PMC8671537; doi:10.1038/s41467-021-27369-3)
Supplement: Supplementary file 3 — Description of Additional Supplementary Files [file 41467_2021_27369_MOESM3_ESM.pdf]

Description of Additional Supplementary Files

File Name: Supplementary Data 1
Description: Table with GISAID accession numbers used in this study
